# Bioturbation effect of artificial inoculation on the flavor metabolites and bacterial communities in the Chinese Mao-tofu fermentation

**DOI:** 10.1016/j.fochx.2024.101133

**Published:** 2024-01-11

**Authors:** Tongwei Guan, Shiyu Fu, Xiaotian Wu, Hao Yu, Ying Liu

**Affiliations:** a College of Food and Biological Engineering, Xihua University, Chengdu 610039, China; b Hanyuan County Xige Mao-tofu Products Factory, Hanyuan 625300, China

**Keywords:** Mao-tofu, Amino acids, Volatile profile, Bacterial community, PacBio sequencing

## Abstract

A comparison between artificially inoculated Mao-tofu (CC) and naturally fermented Mao-tofu (MM) indicated that artificially adding *Mucor plasmaticus* to Mao-tofu dramatically enhanced the essential amino acid (EAA) content, as well as umami and sweet amino acids. Gas chromatography-tandem mass spectrometry (GC–MS/MS) analysis revealed that phenol (3.226 μg/g), 1-octen-3-ol (5.031 μg/g), ethyl heptanoate (1.646 μg/g), and indole (3.422 μg/g) were the key flavor components in Mao-tofu. Unlike MM, CC displayed a substantial increase in esters and a considerable decrease in foul odor substances, including sulfur-containing compounds and indole. *Lactococcus raffinolactis*, *Enterobacter* sp. 638, and *Streptococcus parauberis* KCTC 11537 represented the key bacterial species altering the amino acids and flavor of Mao-tofu according to PacBio single-molecule real-time (SMRT) sequencing and correlation analysis. This study presents the technical feasibility of artificially inoculating Mao-tofu to regulate the core bacterial communities and control the quality of fermented soybean products.

## Highlights

• Biological interference improved the content and abundance of amino acids, and promote the formation of flavor.
• PacBio sequencing was used for the first time to analyze the bacterial community of Mao-tofu.
• Low-abundance bacteria contributed significantly to the flavor of Mao-tofu.

## Introduction

Fermentation is a method of converting organic matter into products via the metabolic activity of microorganisms and biocatalysts. Fermentation is one of the oldest food processing techniques and uses microbial action to produce various food products (Caplice & Fitzgerald, 1999). Fermented soybean products, such as Mao-tofu, natto, stinky tofu, tofu milk, soybean paste, tempeh, and soy sauce, account for a large portion of the fermented foods consumed in China. Mao-tofu is highly popular with consumers due to the specific nutritional benefits and a rich taste provided by microbial participation. Fermented soybean products can be divided into two types based on the fermentation strains: bacterially fermented and moldy soybean products. Like traditional moldy curd, moldy soybean products are manufactured via the fermentation of *Mucor* on a tofu substrate to form a cheese-like product, often referred to by Westerners as Oriental cheese (Han, Rombouts, & Nout, 2001).

Hanyuan County is located in the southern section of Ya'an City, Sichuan Province, with an annual sunlight average of 1450 h and a temperature of 22 °C, providing ideal conditions for producing a unique gourmet Mao-tofu. Mao-tofu is a moldy fermented soybean product in which the mold spores form a white mycelium on the product surface after 48 h of germination. The mycelium becomes denser with extended fermentation time as it proliferates across the tofu surface, consequently becoming known as Mao-tofu. This product derives its distinct flavor from several metabolic events triggered by microorganisms throughout the fermentation process. Enzyme degrades the primary molecules like proteins, lipids, and carbohydrates in soybeans into small molecules such as peptides, amino acids, fatty acids, and simple sugars during microbial fermentation. Since fermentation occurs in an open environment, a broad range of microorganisms are involved, the roles of which vary. The pace of the fermentation and maturation of raw materials, the color of the completed product, and the taste of freshness are all affected by different types of microorganisms during the fermentation process. The small molecules produced via decomposition can also be an important indicator for evaluating the flavor of fermented soybean products (Yang, Tan, & Kan, 2020).

The timed Selective Reaction Monitoring (SRM) data acquisition function of chromatography-mass spectrometry is capable of simultaneously conducting component separation and structural analysis. Tandem-mass spectrometry (MS/MS) significantly reduces the need for sample determination and is capable of simultaneously detecting up to the pg level and screening individual compound before further separation, saving time and costs (Choudhury et al., 2022).

Recent studies have utilized next-generation sequencing (NGS) to quantify and analyze environmental microbiota. Shorter sequences with relatively low taxonomical resolution have limited the classification of microorganisms in the community at the genus level. Third-generation Pacific Biosciences (PacBio) single-molecule, real-time (SMRT) sequencing generates long reads, increasing classification sensitivity and accuracy and allowing for relatively high taxonomic resolution at the species level (Du, Pan, & Chen, 2017).

Mao-tofu is mainly produced via environmental microbial fermentation. Commercial Mao-tofu (BB) is traditionally produced by family workshops and small factories, and then sold in the market. The research on Mao-tofu mainly focuses on the separation, screening, and optimization of protease production conditions by *Mucor*, while there is currently no relevant analysis on the changes in bacterial nutrition as well as volatile components between artificially inoculated and naturally fermented Mao-tofu. Yan et al. (2020) used PCR-DGGE to track the evolution of bacterial diversity in fermented Mao-tofu. However, the ecological makeup and structural properties of the microorganisms in the samples remain unknown. Consequently, using high-throughput sequencing analysis to study microbial diversity, population structure, interaction relationships, and their relevance to environmental factors is a vital step toward Mao-tofu industrial production (Gao, Zhao et al., 2023). This study employs gas chromatography-tandem mass spectrometry (GC–MS/MS) technology to detect and evaluate the volatile compounds in the Mao-tofu produced via both processes. For the first time, the effect of artificial inoculation on the nutrients and volatile components in Mao-tofu is analyzed, while PacBio SMRT sequencing technology is used to further compare the bacterial communities in the two types of Mao-tofu. The functional bacterial communities that may be involved in Mao-tofu fermentation and the core bacterial genera correlated with the nutrient composition and volatile flavor compounds are also explored. The results can increase the understanding of the bacterial composition during Mao-tofu production and help control the quality of the related products effectively. Our study presents the technical feasibility and prospects of regulating flavor compounds via artificial inoculation.

## Materials and methods

### The fermentative production of Mao-tofu and sampling

**Fig. 1:**
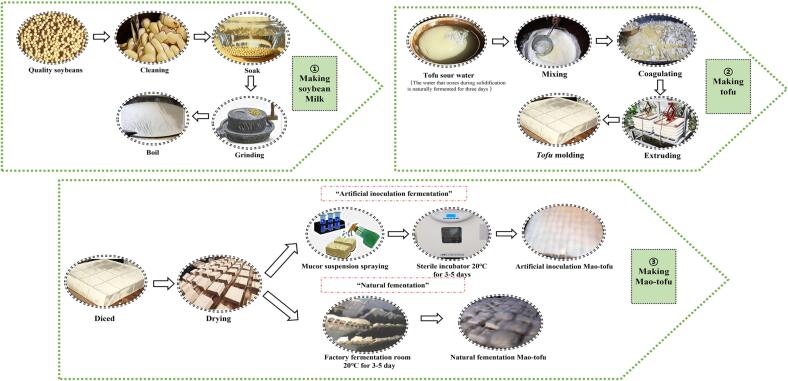
The manufacturing process of natural fermented Mao-tofu and artificial inoculation of Mao-tofu.

Fig. 1 depicts the Mao-tofu production process. The soybeans were purchased from a local food grain market, selected, soaked, grinded, and boiling into soymilk, ordering sour water. After waiting for about ten mintes to coagulate into bean curd, pour it into the covered mold, squeeze out excess slurry, let it stand for a while, and then loosen the mold to make bean curd, cutting into 5 cm × 5 cm × 3 cm bean curd pieces. The samples were divided into two parts, one of which was sprayed with 20 ml spore suspension with a concentration of 1.6 × 10^6^ cfu/ml, placed in an aseptic incubator for fermentation and labeled inoculated Mao-tofu (CC), while the other was naturally fermented in the fermentation room and labeled naturally fermented Mao-tofu (MM). The two types of Mao-tofu were fermented at 20 °C at a humidity of 80 %, and the mature Mao-tofu was collected after 3–5 d (Zhao & Zheng, 2009). The Mao-tofu and sour water were obtained from the Yu Hao Jiuxiang Mao-tofu workshop in HanYuan County, Ya'an City, Sichuan Province, China (samples were transferred to the laboratory on ice). The spore suspension was made from a high protein-producing strain was isolated from the Mao-tofu samples, and the sequence analysis of the isolate was performed using BLAST engine (NCBI) as *Mucor plasmaticus*, upload the gene sequence to NCBI, Sequence ID: OL989887. Its basic culture medium is PDA (Potato 200 g, glucose 20 g, agar 20 g, peptone 5 g, potassium dihydrogen phosphate 3 g, magnesium sulfate 1 g, water 100 ml with natural pH).

### Assessing the nutritional components and textural properties

The nutritional composition indicators of MM, CC, and BB were examined simultaneously, and the experimental data were averaged after three parallel determinations. Mixed 5 g (accurate to 0.0001) of tofu with 50 ml of distilled water, grinded it in a sterile mortar, then filtered it through a layer of cotton cloth, and put the filtrate into a small beaker, the pH was determined using a PHS-320 pH meter (Shanghai, China). The yield was calculated based on the weight of the fresh tofu that could be produced from 100 g of soybeans. The moisture level was determined using the atmospheric pressure drying method, while the ash content was ascertained using the direct ash method. The moisture activity was measured with a moisture activity meter LabSwift (Novasina, Switzerland), while the water-soluble protein level was measured via the Kjeldahl method. The total protein content was calculated by multiplying the nitrogen content by 6.25 (AOAC, 2000). The color differences were identified using a CR-400 colorimeter (Konica Minolta, Japan), while the colors were expressed as L* (brightness) and b* (yellowness) values. The vitamin C (Vc) content was determined via the colorimetric method using 2,4-dinitrophenyl hydrazine (Reddy & Srinivasan., 1948). Additionally, the textural analysis was performed using a textural instrument (TA-XTPLUS, Stable Micro System, Britain), and the textural properties, including hardness, cohesiveness, gumminess, resilience, adhesiveness, and chewiness, were determined using the texture profile analysis (TPA) secondary compression method (Nishinari, Kohyama, Kumagai, Funami, & Bourne, 2013), where the samples were cut into 3 cm × 3 cm × 1 cm cubes and compressed twice using a P/36R probe. The TPA parameters were as follows: a pre-test speed of 2.0 mm/s, an in-test speed of 1.0 mm/s, a post-test speed of 2.0 mm/s, a correction height of 30 mm, a downward compression distance of 4 mm, PPS: 200, time: 5.00 s, and trigger force: 4 g.

### Analysis of the amino acid content

The amino acids in the samples were determined using an automated amino acid analyzer (S-4330D, Sykam, Germany). The experimental method is briefly described as follows: 10 ml 55 % HCl is added to 0.1 g of hairy tofu sample, hydrolyzed at 110℃ for 24 h, and then the sample is neutralized with NaOH at pH 2.2. After centrifugation at 79,920 g for 10 min, the sample was filtered through a 0.22 μm filter membrane. Then the protein supernatant was quantitatively analyzed according to the retention time and the peak area relative to the amino acid standard (Zhu & Wu., 1988).

#### Evaluation method

##### Calculating the amino acid ratio coefficient (RC)

Using the WHO/FAO essential amino acid (EAA) model, the amino acid ratio (RAA), RC, and ratio coefficient score (SRC) of the EAA in the samples were calculated based on the theory of amino acid balance (Zhang et al., 2012).

$$\mathrm{RAA}=\frac{\mathrm{sample}}{\mathrm{The}\;corresponding\;EAA\;content\;in\;WHO/FAO}\left(\mathrm{mg}/g\right)$$

$$\text{RC=}\frac{\text{RAA}}{\text{Mean of RAA}}$$

RC > 1 indicates a relative EAA excess, and RC < 1 denotes a relative EAA deficiency. The first limiting amino acid represents the minimum RC.

SRC=100-CV×100

CV is the coefficient of variation of RC, CV = standard deviation/mean.

##### Fuzzy identification method

The Lang's distance method was used to determine the closeness μ (α, u_i_) of the protein ui and the model protein (egg protein) α, which was calculated as:

$$\mu \;\left(\alpha ,\;ui\right)\;=1-c=\sum_{k=1}^{7}\frac{\left|\alpha_{k}-u_{\mathrm{ik}}\right|}{\left|\alpha_{k}+u_{\mathrm{ik}}\right|}$$

where c is an adequately chosen constant and defined as 0.09 to obtain the calculation results in the interval [0, 1].

#### Amino acid TAV analysis

TAV is the ratio of flavor substances in samples to their corresponding threshold, which is widely used in the study of various food flavors. When TAV is greater than 1, taste active substances have a significant contribution to the overall taste of the sample, and its value is directly proportional to the contribution. On the contrary, when TAV is less than 1, the contribution of taste active substances to the overall taste of the sample is very small. The different taste characteristics of the various free amino acids were classified according to the TAV values. The taste contribution was directly related to the threshold of the amino acids (Wang et al., 2019). The TAV value refers to the ratio of the threshold value of the amino acid to its content (mg/100 g).

### Determining the volatile flavor substances

#### Sample extraction

The Mao-tofu samples (3 g) were mixed with distilled water at a ratio of 1:10 in a 50 ml centrifuge tube and ultrasonically pre-treated for 30 min. Next, the mixture was centrifuged in a high-speed refrigerated centrifuge at 4 °C and 10,000 g for 10 min to obtain 5 ml supernatant, which was added to the headspace bottle (20 ml, Agilent) along with 100 μL 1.4 mg/L 2-octanol as the internal standard. The volatile compounds were extracted via a StableFlex fiber (50/30 μm DVB/CAR/PDMS StableFlex, Supelco Co., Bellefonte, PA, USA) at 60 °C for 45 min, store SPME in GC sampler for 3–5 min (Dennison et al., 1978).

#### GC–MS/MS parameter settings

Gas chromatography-tandem mass spectrometry (GC–MS/MS) was used for analysis (GCMS2020NX, Shimadzu, Japan). HP-INNOWax column (34 m × 0.25 mm × 0.25 μm) and a DB-WAX column (1.4 m × 0.25 mm × 0.25 μm). The analysis conditions were as follows: inlet temperature 230 °C, column head pressure 49.14 psi, carrier gas flow rate 1.3 ml/min. The oven temperature was held at 40 °C for 2 min and then increased to 230 °C at a rate of 6 °C/min (Chamkasem, Lee, & Harmon, 2016).

#### Identification and semi-quantitative analysis of the volatile compounds

The flavor components of the Mao-tofu were analyzed using the Canvas Version 1.8 software (Canvas, China), a comprehensive two-dimensional chromatographic data processing system (Yan & Dong, 2019). The mass spectrum data was compared with the NIST 05a MS database to determine the compositions of the volatile flavor compounds in the Mao-tofu samples. The semi-quantitative internal standard method was used to determine the volatile flavor substance levels in the Mao-tofu samples by comparing their peak areas with the 2-octanol internal standard. The amount of each compound was calculated using the following formula:

$$C\left(\mu g/g\right)=\;\times Cis\left(\mu g/g\right)$$

C refers to the relative concentration of analytes, Cis denotes the concentration of the internal standard in samples, Ac represents the peak areas of the analytes, and Ais signifies the peak area of the internal standard.

### PacBio SMRT sequencing

#### Sample preparation and DNA extraction

The Mao-tofu samples were thawed, and the DNA was extracted using a DNA extraction kit (OMEGA Bio-tek Inc., Norcross, US) according to the instructions of the manufacturer. The integrity, purity, and concentrations of the DNA samples were determined using 1 % agarose gel electrophoresis and a microscale ultraviolet spectrophotometer. High-quality DNA was stored at −20 °C until evaluation for no longer than one month (Mosher et al., 2014).

#### Amplification of the full-length 16S rRNA and PacBio SMRT sequencing

The full-length sequences of the bacterial 16S rRNA genes were selected as the target amplification fragments. The extracted DNA was used as a template, and the 16S full-length universal primer was used for PCR amplification (upstream primers, 5′-AGAGTTTGATCMTGGCTCAG-3′, downstream primers, 5′-ACCTTGTTACGACTT-3′). An identification tag barcode containing 16 bases was added to both ends of all primers to distinguish different samples in the same library. The whole PCR solution was prepared by mixing 1.5 μL of 20 ng/μL DNA, 1.5 μL of 10 μmol/L upstream primers, 1.5 μL of 10 μmol/L downstream primers, 25 μL of 5 U/μL polymerase mixture (KAPA HiFiTM HotStart ReadyMix PCR Kit, US), and 20.5 μL sterilized ultra-pure water. The droplets produced by each sample were transferred to a 96-well plate, and PCR amplification was performed using the EvaGreen program: 95 °C for 3 min, 98 °C for 20 s, 62 °C for 15 s, 72 °C for 45 s, 30-cycles, followed by 72 °C for 1.5 min. The PCR products were purified by adding an equal volume of Gnome DNA Clean magnetic beads. The DNA concentration was measured using a Qubit dsDNA HS Assay Kit (Thermo Fisher Scientific, Waltham, MA). The samples with DNA concentrations exceeding 20 ng/μL were used to construct the DNA library (Pacific Biosciences SMRT bell^TM^ Template Prep Kit 1.0) and perform sequencing using a PacBio SMRT RSⅡ instrument *P*6-C4 reagent computer at movie times of 240 min in CCS mode (Mosher, Bernberg, Shevchenko, Kan, & Kaplan, 2013). All raw sequencing data were submitted to the Sequence Read Archive (SRA) of the National Center for Biotechnology Information (NCBI; Bethesda, MD, USA) under the project accession number: PRJNA791188.

#### Sequence processing

To extract high-quality sequences, the quality of the original sequences was controlled using RS_Reads Ofinsert. 1. The quality control standard demanded no less than five cycles, a minimum prediction accuracy of 90, and minimum and maximum inserted fragments of 1400 bp and 1800 bp, respectively (Mosher, Bernberg, Shevchenko, Kan, & Kaplan, 2013). The subsequent high-quality sequences were divided among the corresponding samples according to the barcode. Next, the barcode and primers of each sequence were removed before subsequent bioinformatics analysis via the QIIME (V1.7.0) platform.

#### Bioinformatics analysis

The sequences were aligned using PyNAST, while UCLUST was used to divide the operational taxonomic units (OTUs) according to 100 % clustering of the sequence identity to obtain the representative sequences. The OTUs were then classified according to 97 % similarity, and chimeric OTU sequences were removed via Vsearch (Bottacini & van Sinderen, 2021). The Ribosomal Database Project (RDP, Release 11.5), Greengenes (version 13.8), and Silva (version 128) databases were used to homologically compare the representative OTU sequences and determine the taxonomic status of each (Westbrook et al., 2015). The sequence number of each sample was standardized to determine the alpha and beta diversity. The rarefaction and Shannon diversity curves were used to determine whether the sequencing depth represented the bacterial composition in the sample. The abundance and diversity of the corresponding communities in the samples were evaluated by calculating the OTU number and Shannon diversity indexes at the same sequencing depth.

### Statistical analysis

All experiments were performed in triplicate, while the plot data were processed using Excel 2020 and Origin 2021. SPSS 21.0 software was used to analyze the differences between the Mao-tofu samples via ANOVA and post hoc tests. The differences between the means of the Mao-tofu groups were considered significant at *P* < 0.05 and were determined using principal component analysis (PCA).

## Results and discussion

### Nutritional and textural characteristics of the Mao-tofu

The nutritional composition results are shown in Table 1. The MM and CC samples displayed a somewhat higher moisture content than BB. The high moisture content was partially due to the significant protease secretion by *Mucor*, breaking the proteins down into small molecules (Alves, Campos-Takaki, Okada, Ferreira-Pessoa, & Milanez, 2005), which increased the uniformity of the tofu and retained moisture. Tofu with a high moisture content usually indicates a higher yield. Therefore, the results of this study showed that the Mao-tofu yield exceeded BB (Cheng, J. K., 2007). The protein content of MM and CC was 25.08 % and 24.07 %, respectively, with Vc levels of 1.19 mg/100 g and 1.02 mg/100 g, which were higher than BB (*P* < 0.05). Fermented soybean products typically retain the rich nutrients of soybeans while significantly increasing the nutrient content through a series of microbial fermentation. Therefore, the Mao-tofu products exhibited a higher nutritional value than BB. CC displayed a lower water activity (A_w_) value than MM, indicating more effective water retention and microbial utilization in CC than MM. The artificial inoculation of *Mucor* strains in aseptic conditions ensures fermentation train specificity and avoids interference from other strains (Grant & Mertens, 1992). Creamy white is the optimal color for commercial tofu. A lower L* value induces darker colored tofu, while a higher b* value increases the yellowness. Despite the dense layer of white *Mucor* covering the Mao-tofu exterior, the *Mucor* spores are usually chartreuse, resulting in a yellowish color and reducing the L* of the product. Polysaccharide, cellulose, and phytic acid changes in the tofu due to microbial fermentation may also affect the Mao-tofu color (Koca, Karadeniz, & Burdurlu, 2007). Furthermore, fermentation affects the texture and refractive index of tofu, impacting its color (Rana, Pradhan, & Mishra, 2019).

**Table 1:** Physicochemical results of the three samples.

Sample	BB	MM	CC
Moisture /%	75.9 ± 1.53^a^	80.4 ± 1.15^b^	83.3 ± 1.10^c^
Yield (g/100 g)	133.5 ± 12.1^a^	212.4 ± 12.4^b^	197.1 ± 11.3^b^
Protein /%	18.40 ± 1.33^a^	25.08 ± 3.41^c^	22.07 ± 2.24^b^
Aω	0.94 ± 0.02^b^	0.88 ± 0.04^b^	0.72 ± 0.14^a^
pH	6.01 ± 0.01^a^	7.83 ± 0.18^b^	7.28 ± 0.08^b^
L *(o)	81.38 ± 11.87^a^	80.16 ± 12.58^a^	85.03 ± 13.72^b^
L *(m)	90.09 ± 10.89^b^	79.83 ± 10.22^a^	88.19 ± 10.04^b^
b *(o)	20.89 ± 3.30^a^	25.60 ± 2.36^b^	19.34 ± 1.21^c^
b *(m)	20.33 ± 0.07^a^	19.50 ± 0.71^c^	19.02 ± 0.23^c^
Crude Ash (g/100 g)	0.89 ± 0.30^a^	1.10 ± 0.04^b^	1.31 ± 0.01^b^
Solubility sugar (g/100 g)	0.48 ± 0.62^a^	1.02 ± 0.15^d^	0.59 ± 0.71^b^
Vc(mg/100 g)	0.83 ± 0.22^a^	1.19 ± 0.09^b^	1.02 ± 0.43^a^

The physical and chemical properties and color of three samples are the mean ± SD from triplicates. Different letters within a column indicate significant differences (P < 0.05).

The TPA results of the Mao-tofu are shown in Fig. S1. The chewiness, hardness, and elasticity values of CC were slightly higher than MM and significantly exceeded those in BB, while MM displayed the maximum viscosity, which corresponded with what was visible to the naked eye. Artificial inoculation intensified the fermentation process. CC presented a higher number of small pores with a more continuous and homogeneous structure, allowing it to accommodate more proteins and other soluble substances, which increased the hardness, chewiness, and elasticity. MM may contain a large number of dietary fibers such as cellulose, soy polysaccharides, and other insoluble substances due to insufficient fermentation, weakening the interprotein connections that lead to a looser network structure, which decreases the hardness, elasticity, and chewiness of MM gels (Yan & Dong, 2019).

### Identification and quantification of amino acids

The Mao-tofu fermentation process generally enhanced all 17 amino acids (Table S1). The total amino acid (TAA) content per 100 g of CC and MM was 76.26 g and 78.07 g, respectively, increased by 39.3 % and 42.69 %, respectively, compared to BB. MM and CC were rich in essential amino acids (EAA) with EAA/TAA % values of 43.42 % and 48.49 %, respectively, and the latter 5.07 % higher than the former. Moreover, the EAA/non-essential amino acid (NEAA) levels were 76.74 % and 94.14 % in MM and CC, respectively, with the latter 17.40 % higher than the former. Although no divergence was evident between the TAA content of CC and MM, variation in the EAA to NEAA ratio was apparent in CC relative to MM, which could be due to the artificially inoculated *Mucor plasmaticus* releasing a higher amount of protease to obtain more key amino acids. However, many bacteria in Mao-tofu may also produce a variety of amino acids, enriching the amino acid composition in the product and improving its flavor. The five principal amino acids in CC and MM were α-aminoglutaric acid (Glu), aspartic acid (Asp), isoleucine (Ile), lysine (Lys), and valine (Val). The Lys and proline (Pro) levels in BB were 3.26 g/100 g and 2.37 g/100 g, respectively. This content increased significantly in the Mao-tofu, with the highest Lys level in CC at 7.35 g/100 g and the highest Pro level in MM at 5.29 g/100 g. Lys and Pro are vital for maintaining healthy skin and connective tissue growth since they assist the body in breaking down proteins and can also be transformed into hydroxyproline and hydroxylysine to aid collagen formation (Ramirez-Jimenez, Garcia-Villanova, & Guerra-Herna, 2004). CC displayed increased Val and Ile levels. Val interacts with Ile and leucine (Leu) to promote normal bodily growth, repair tissue, regulate blood sugar and provide sufficient energy (Baker et al., 1996).

If the amino acids levels in fermented food are close to or correspond with the WHO/FAO amino acid, they are deemed suitable for human physiological activity and display adequate nutritional value (Vinayashree & Vasu, 2021). According to the RAA, the Val level was highest in CC at 7.69-fold that of the WHO/FAO requirement pattern and could be used as a supplementary source for most cereal proteins in which Val represented the first limiting amino acid (Table S2). MM exhibited the highest threonine (Thr) content at 6.03-fold that of the WHO/FAO requirement pattern and could be used as a supplementary source for most cereal proteins in which Thr represented the first limiting amino acid (Table S2). The RC values revealed that phenylalanine (Phe) and tyrosine (Tyr) represented the limiting amino acids in CC, MM, and BB, denoting the first determinants of protein utilization in these groups. The similarity of CC, MM, and BB to egg protein (standard protein) was determined using the fuzzy identification method (Table S3). The proximity of CC tends to 1, indicating that a more substantial positive contribution by the EAA in CC in terms of the physiological role of amino acid balance increased the nutritional value of the Mao-tofu.

The amino acid TAV values of the three samples are shown in Table S4. Some amino acids exhibited TAV values exceeding 1 in CC and MM, such as sweet amino acids (Thr, alanine (Ala), and Gly), umami amino acids (Glu and Asp), and bitter amino acids (Leu, Val, His, and dl-methionine (Met)), positively affecting the sweetness, umami, and bitterness of Mao-tofu. Furthermore, the TAV values of the amino acids (Val, Ile, and His) that affected bitterness in CC decreased moderately, while those affecting umami (Glu and Asp) displayed a slight increase, indicating that artificial intervention may change the Mao-tofu flavor to a certain extent by adjusting the amino acid levels. In addition to their compositions and concentrations, the taste of amino acids is also affected by environmental conditions, such as pH, temperature, and technological processes (Iwaniak et al., 2016).

### Identification and difference analysis of volatile compounds

**Fig. 2:**
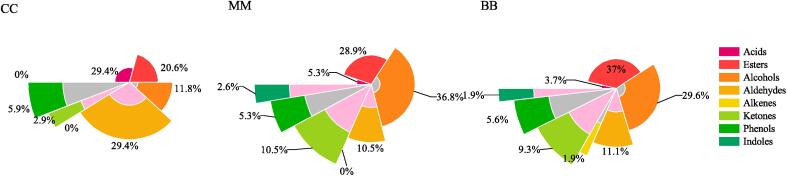
Comparison of total volatile compounds of three samples.

Fig. 2 shows the overall distribution of the flavor compounds in the three samples. Here, 21 volatile components were identified in BB, including six acids, five aldehydes, four alcohols, three esters, one phenol, one ketone, and one sulfur compound. MM contained 43 volatile components, including 15 alcohols, eight esters, four ketones, three acids, three aldehydes, three phenols, three sulfur compounds, and two heterocycles. Furthermore, 47 flavor compounds were identified in CC, including 20 esters, 9 alcohols, 3 ketones, 3 acids, 3 aldehydes, 2 phenols, 3 sulfur compounds, and 2 heterocycles. All the components in the three samples represented food flavors permitted by the Flavor and Extract Manufacturers Association of the United States (FEMA).

The types and concentrations of the different flavor substances with a relative percentage surpassing 1 % in the three samples are shown in Table S5. Alcohols and esters accounted for the highest proportion in the Mao-tofu. The most abundant alcohols in MM included 1-octen-3-ol (6.374 μg/g), ethanol (2.816 μg/g), 1-octanol (2.215 μg/g), *n*-propanol (0.928 μg/g), and 3-octanol (0.882 μg/g), of which 1-octen-3-ol was produced by the oxidative decomposition of linoleic acid with the participation of fungi, emitting a lavender and licorice fragrance (Gao, Zhao et al., 2023). Furthermore, 1-octanol presented a strong oily, citrus scent, while *n*-propanol induced a characteristic ripe fruit flavor, these alcohols endow Mao-tofu with mellow aroma. The highly abundant esters in CC imparted aroma characteristics of fruit, wine, and honey to the Mao-tofu. The aroma elicited by ethyl palmitate (1.646 μg/g) was fruity and creamy, while a grape, raspberry, cheesy aroma resulted from ethyl heptanoate (1.646 μg/g). Ethyl caproate (1.481 μg/g) provided an aroma comprising fruit and wine, while that of ethyl acetate (1.342 μg/g) corresponded to pineapple and rose (Zhang et al., 2022).

The esters were significantly more abundant in CC than in MM. The ethanol content in CC was also considerably higher. Esters are primarily produced via the action of fatty acid synthase on acetyl coenzyme A (acetyl-CoA) and the corresponding higher alcohols. Acetyl-CoA is also a precursor for synthesizing fatty substances, resulting in competition between ester and lipid metabolism. Carbohydrate metabolism is significantly more abundant than lipid metabolism during Mao-tofu fermentation, which is likely why Mao-tofu has such a high level of esters (Lee, JW, &Trinh, CT, 2020). Moreover, some studies revealed that curd with added ethanol during the late stage of fermentation contained 61 compounds, showing a notable increase in the number of esters, while only 55 compounds were detected in curd without ethanol addition (Hwan & Chou, 2000).

Several other highly abundant chemicals in MM and CC were also examined. Benzaldehyde presented a bitter almond, cherry, and nutty aroma, while phenol provided a strong medicinal (Zhang et al., 2022), spicy and smoky odor, reaching 3.226 μg/g and 2.265 μg/g in MM and CC, respectively. The butyric acid content was higher in the Mao-tofu than in BB. Butyric acid, a short-chain fatty acid, can be generated in tofu via microbial carbohydrate fermentation. It is also believed that butyric acid efficiently prevents tumor formation by blocking histone deacetylase (Orchel, Dzierzewicz, & Wilczok, 2005).

Moreover, flavor compounds, such as dimethyl trisulfide and dimethyl tetrasulfide, provide strong onion and radish scents, while 2-amylfuran and indole present an animal fecal odor. Although these substances were previously reported as characteristic aroma components of the odor in fermented stinky tofu, this study indicated that their levels were reduced in CC. This phenomenon could be attributed to the impact of the different manufacturing processes on the Mao-tofu texture. Some studies have revealed changes in the textural parameters of tofu, such as the hardness and viscosity, can produce malodors, volatile sulfides, and indoles (Ma, Wang, & Cheng, 2013). To summarize, artificial inoculation can reduce the unpleasant odor in Mao-tofu while retaining the original flavor, enhancing consumer acceptance.

### The bacterial community structure of artificially and naturally fermented Mao-tofu

After PacBio SMRT sequencing, 145,276 high-quality 16S rRNA gene sequences were obtained, with an average sequencing quantity of 242,12 sequences per sample. After 100 % similarity cluster analysis, 104,811 representative reads were obtained. Furthermore, 85,603 OTUs were obtained at 97 % similarity. During this experiment, the rarefaction and Shannon curves were used to evaluate the sequencing volume and biological diversity of the microbiota at the 97 % similarity level (Fig. S2). As the sequencing amount increased, these curves tended to be horizontal in all the samples, indicating that although it was possible to find new bacterial germline types, the current sequencing amount met the requirements for subsequent bioinformatics analysis and effectively revealed the composition of the main flora in the samples.

**Fig. 3:**
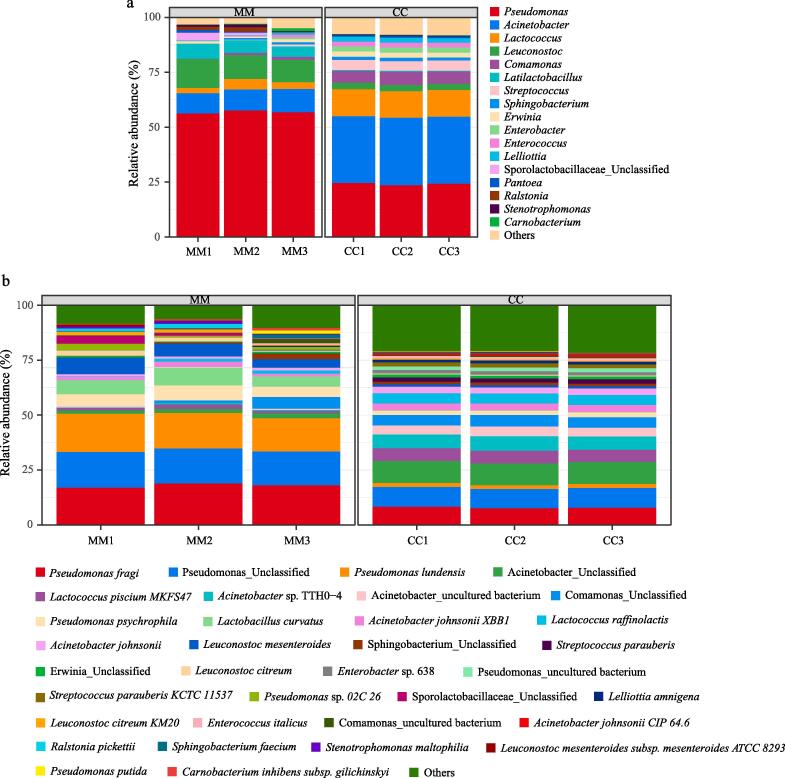
Histogram of bacterial species distribution of artificially inoculated (CC) and naturally fermented (MM) Mao-tofu. a: Relative abundance of bacterial genus, b: Relative abundance of bacterial species.

All bacterial OTUs were classified taxonomically into seven phyla, 11 classes, 25 orders, 47 families, 94 genera, and 318 species. The nine most abundant bacterial genera in the Mao-tofu samples included *Pseudomonas*, *Leuconostoc*, *Lactobacillus*, *Acinetobacter*, *Sporolactobacillaceae*_Unclassified, *Lactococcus*, *Ralstonia*, *Pantoea,* and *Erwinia*, with average contributions of 60.48 %, 13.24 %, 6.71 %, 4.99 %, 3.52 %, 2.44 %, 1.48 %, 1.27 %, and 1.03 % to all bacterial reads, respectively (Fig. 3a). Altogether, these genera accounted for over 90 % of the total bacterial sequences. *Pseudomonas* is abundantly present in the air, soil, water, and food, and typically contributes to the flavor of naturally fermented food (Doulgeraki & Nychas, 2013). Notably, Pseudomonas has decarboxylase activity, and Mao-tofu is rich in amino acids. The amino acids in Mao-tofu are prone to producing biogenic amines under the action of decarboxylase. Excessive intake of biogenic amines in the human body can lead to discomfort and even death (Gao et al., 2022). Therefore, the abundance of Pseudomonas in Mao-tofu is worth further research. *Acinetobacter* contains many probiotic strains that improve intestinal flora dysbiosis in obese mice on high-fat and high-sugar diets, where the intestinal microbiota plays a critical role in host energy homeostasis (Kong et al., 2019) (*P* < 0.05).

A high abundance of dominant microorganisms is often considered an essential component of food fermentation. As shown in Fig. 3b, MM contained a total of 298 bacteria at the species level, of which those with a relative abundance above 1 % included *Pseudomonas fragi* (18.89 %), *Pseudomonas*_Unclassified (16.25 %), *Pseudomonas lundensis* (15.71 %), *Leuconostoc mesenteroides* (7.57 %), *Lactobacillus curvatus* (6.41 %), *Pseudomonas psychrophila* (5.28 %), *Leuconostoc citreum* (2.23 %), *Acinetobacter*_Unclassified (1.46 %), *Acinetobacter johnsonii* XBB1 (1.44 %), and *Lactococcus piscium* MKFS47 (1.03 %). These species accounted for over 76.27 % of the total bacterial sequences. CC contained a total of 152 bacteria at the species level, of which those with a relative abundance exceeding 1 % included *Acinetobacter*_Unclassified (9.92 %), Pseudomonas_Unclassified (8.70 %), *Pseudomonas fragi* (7.61 %), *Acinetobacter* sp. TTH0 − 4 (6.62 %), *Lactococcus piscium* MKFS47 (5.74 %), *Comamonas*_Unclassified (4.79 %), *Acinetobacter johnsonii* XBB1 (3.26 %), *Pseudomonas psychrophila* (1.96 %), *Pseudomonas lundensis* (1.88 %), and *Leuconostoc mesenteroides* (1.02 %). These species accounted for over 51.5 % of the total bacterial sequences. The abundance of *Pseudomonas fragi*, *Pseudomonas*_Unclassified, and *Pseudomonas lundensis* in CC were significantly lower than in MM, while that of *Acinetobacter* sp. TTH0-4 and *Acinetobacter johnsonii* XBB1 increased substantially. This variation may be caused by the aseptic conditions of artificial inoculation that block the participation of some *Pseudomonas*. Recent research attributes some of this variation to niche pre-emption between *Acinetobacter* and *Pseudomonas* strains (Cheong et al., 2021) (*P* < 0.05).

**Fig. 4:**
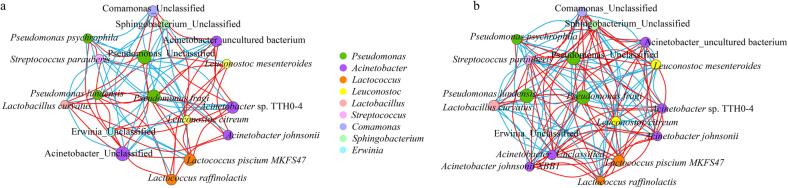
Horizontal correlation network diagram of Mao-tofu, a: naturally fermented, b: artificially inoculated fermented;

The network diagram shows a substantial association between the different bacterial groups of CC and MM (Fig. 4). *Pseudomonas*, *Acinetobacter*, *Leuconostoc*, and *Lactococcus* demonstrated highly positive relationships with other genera in CC and MM. *Lactococcus*, *Acinetobacter*, *Pseudomonas*, and *Kurthia* have been identified as the primary genera contributing to the flavor and nutritional components in food products, such as cheese, curd, soy sauce, wine, teae, and vinegar (Dongmo et al., 2017). At the species level, *Pseudomonas*_Unclassified (degree = 14) connected most of the species in the bacterial network diagram of CC (Fig. 4a), followed by *Acinetobacter* Unclassified (degree = 11). Similar patterns were observed in MM (Fig. 4b), demonstrating that *Pseudomonas* played an essential role in both CC and MM. Furthermore, MM displayed a considerably larger number of species associated with *Acinetobacter*_Unclassified, *Lactococcus piscium* MKFS47, *Comamonas*_Unclassified, and *Sphingobacterium*_Unclassified than CC.

### The correlation of the bacterial community with the amino acids and volatile compounds in Mao-tofu

**Fig. 5:**
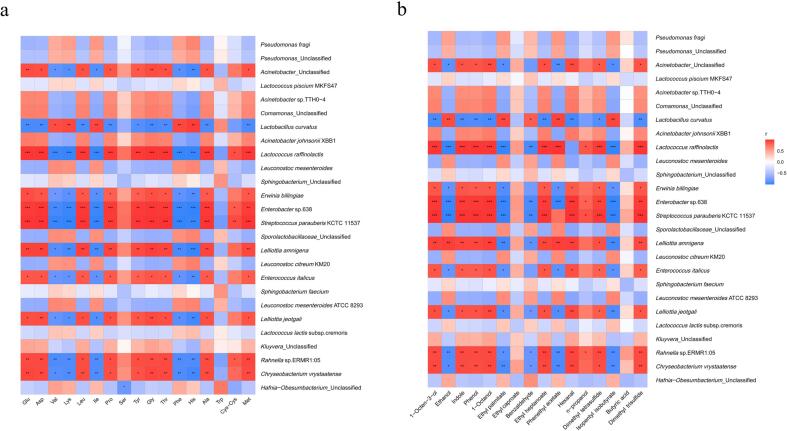
Correlation analysis of bacterial communities with amino acids and volatiles. a: Heatmap of the correlation between amino acids and bacterial communities of Mao-tofu, *represents the correlation between amino acids and species (***: *P* < 0.01, **: *P* < 0.01, *: *P* < 0.05). b: Heatmap of the correlation between flavor compounds and bacterial communities of Mao-tofu, the figure shows the high levels of flavor compounds in all sample, * represents the correlation between flavor compounds and species (***: *P* < 0.001, **: *P* < 0.01, *: *P* < 0.05).

A correlation heatmap was used to visualize the association between the bacterial species and amino acids in the samples, as shown in Fig. 5a. *Lactococcus raffinolactis*, *Enterobacter* sp. 638, and *Streptococcus parauberis* KCTC 11537 showed significantly positive correlations with Gly, Ala, Thr, Glu, Asp, and Leu, where most presented sweet and umami flavors while revealing remarkably negative relationships with Val, Ile, Phe, His, and Cys where most displayed a bitter flavor. This observation suggests that *Lactococcus raffinolactis*, *Enterobacter* sp. 638, and *Streptococcus parauberis* KCTC 11537 may significantly influence the flavor of Mao-tofu, rendering the overall flavor sweet and umami rather than bitter. *Acinetobacter*_Unclassified, *Erwinia billingiae*, *Lelliottia amnigena*, *Enterococcus italicus*, *Leuconostoc mesenteroides* ATCC8293, *Rahnella* sp. ERMR1:05 and *Chryseobacterium vrystaatense* exhibited a markedly positive correlation with Tyr and Met. Some studies have indicated that the bacterial breakdown of Tyr and Met can yield odoriferous chemicals, such as indoles and sulfur compounds, implying that the generation of disagreeable odors in the Mao-tofu may be related to these strains (Muszynska, & Sulkowska-Ziaja, 2012).

The association analysis of the 16 volatile compounds significantly affecting the aroma of Mao-tofu with strains showing a relative abundance higher than 0.1 % in the bacterial communities is shown in Fig. 5b. The heatmap information revealed that *Lactococcus raffinolactis*, *Enterobacter* sp. 638, *Streptococcus parauberis* KCTC11537, *Leuconostoc mesenteroides*, *Chryseobacterium vrystaatense*, and *Hafnia-Obesumbacterium*_Unclassified displayed a significantly positive association with the primary volatile flavor substances in Mao-tofu, including 1-octen-3-ol, indole, phenol, and 1-octanol, suggesting that many bacteria other than *Mucor* are involved in its flavor substance synthesis. *Lactococcus raffinolactis* exhibited a noticeably positive correlation with ethyl heptanoate and phenyl acetate, while some studies claimed that *Lactococcus* was involved in the formation of ethyl caproate, a key flavor compound in strong-flavored Chinese Baijiu (Gao, Zhao et al., 2023). Consequently, *Lactococcus* may be conducive to ester production in Mao-tofu. *Leuconostoc mesenteroides* demonstrated a significantly positive correlation with ethanol, while *Leuconostoc* relied exclusively on the hexose monophosphate (HMP) pathway for glucose degradation during fermentation due to the lack of aldolase and isomerase in the Embden-MeyerhofParnas pathway (EMP) pathway. Therefore, ethanol represented the primary fermentation product (Kim, Chun, &Han, 2000).

The amino acid and volatile compound production in the Mao-tofu resulted from the synergetic effect of multiple microorganisms throughout the entire fermentation micro-ecosystem. Therefore, the relationship between microbes and metabolites cannot be fully described based on simple correlation analysis alone. Furthermore, the raw materials and processing method utilized when manufacturing Mao-tofu significantly impacted the microbial community structure and product characteristics, requiring further research.

## Conclusions

This study suggests that artificial inoculation improves the texture and color of Mao-tofu and enhances its appearance and taste. This increases consumer acceptance of Mao-tofu compared to MM. The increased EAA content in CC rendered it generally similar to the composition of amino acids in standard proteins and more suitable for human absorption. The types and amounts of flavor substances, such as alcohols, esters, aldehydes, ketones, and phenols, are substantially higher in CC and MM than BB. The primary flavor components in the Mao-tofu include phenol, 1-octen-3-ol, ethyl heptanoate, and indole. Artificial inoculation increases the ester content while reducing the sulfur compounds and heterocycles in the Mao-tofu, resulting in a mellower flavor of CC. *Pseudomonas*, *Leuconostoc*, *Lactobacillus*, and *Acinetobacter* represent the bacterial genera with the highest abundance in Mao-tofu. According to the correlation analysis, *Lactococcus raffinolactis*, *Enterobacter* sp. 638, and *Streptococcus parauberis* KCTC 11537 denote the key bacterial species affecting the amino acids and flavor in Mao-tofu. This paper reflected in the regulation of core bacterial communities. This study provides a scientific foundation for expanding the application of functional fermented soybean products.

## CRediT authorship contribution statement

**Tongwei Guan:** Formal analysis, Data curation, Conceptualization. **Shiyu Fu:** Conceptualization. **Xiaotian Wu:** Data curation. **Hao Yu:** Conceptualization. **Ying Liu:** Supervision.

## Declaration of competing interest

The authors declare that they have no known competing financial interests or personal relationships that could have appeared to influence the work reported in this paper.

## Data Availability

The authors do not have permission to share data.
