# Supplementary material for: Bioturbation effect of artificial inoculation on the flavor metabolites and bacterial communities in the Chinese Mao-tofu fermentation
Source: Food Chem X. 2024 Jan 11;21:101133. doi: 10.1016/j.fochx.2024.101133 (PMC10832485; doi:10.1016/j.fochx.2024.101133)
Supplement: Supplementary data 1 [file mmc1.docx]

**Supplementary data**

Bioturbation effect of artificial inoculation on flavor metabolite and bacterial community in Chinese traditional Mao-tofu fermentation microecosystem

Shiyu Fu^a^, Qingyan Guo^a^, Hao Yu^b^, Han Yang^a^, Yurun Tang^a^, Tongwei Guan^a,⁎^

*College of Food and Biological Engineering, Xihua University, Chengdu, 610039, People’s Republic of China.*

*^b^Hanyuan County Xige Soybean Products Factory, Hanyuan 625300, China.*

**^⁎^Author for correspondence: Tongwei Guan**

Tel/Fax: +86 028 87720552

E-mail: guantongweily@163.com

Submitted to ***Food Chemistry: X***

**Table S1**

Amino acid composition of the three samples (P<0.05).

| Amino acid | | sample (mg/g) | | |
| --- | --- | --- | --- | --- |
|  |  | CC | MM | BB |
| EAA | Thr | 3.65 | 4.22 | 2.17 |
|  | Val | 7.69 | 6.92 | 3.69 |
|  | Met | 0.26 | 0.65 | 0.34 |
|  | Ile | 6.61 | 5.84 | 4.84 |
|  | Phe | 4.38 | 2.13 | 3.22 |
|  | Lys | 7.35 | 6.44 | 3.26 |
|  | Leu | 6.05 | 6.83 | 4.25 |
|  | Trp | 0.99 | 0.87 | 0.63 |
|  | Ser | 5.02 | 5.17 | 3.63 |
|  | Glu | 10.63 | 11.2 | 8.63 |
|  | Pro | 4.87 | 5.29 | 2.37 |
|  | Gly | 3.71 | 4.36 | 1.13 |
| NEAA | Ala | 1.05 | 1.84 | 1.48 |
|  | Cys-Cys | 0.66 | 0.78 | 2.04 |
|  | Tyr | 3.92 | 4.58 | 4.84 |
|  | His | 1.75 | 1.24 | 0.54 |
|  | Asp | 7.67 | 9.71 | 7.65 |
| TAA | | 76.26 | 78.07 | 54.71 |
| EAA/NEAA（%） | | 94.14 | 76.74 | 69.32 |
| EAA/TAA (total) % | | 48.49 | 43.42 | 40.94 |

Note: EAA represent essential amino acid, NEAA represent non-essential amino acids, TAA represent total amino acids.

**Table S2**

Comparison of RAA, RC and SRC of the 3 sample proteins.

| Sample ID | RAA、RC | Thr | Val | Ile | Lys | Leu | Trp | Met + Leu | Tyr + Phe |
| --- | --- | --- | --- | --- | --- | --- | --- | --- | --- |
| CC | RAA | 5.21 | 7.69 | 6.61 | 5.04 | 5.25 | 2.82 | 1.96 | 1.09 |
|  | RC | 1.16 | 1.72 | 1.48 | 1.13 | 1.17 | 0.63 | 0.44 | 0.24^*^ |
|  | SRC | 91.5 | | | | | | | |
| MM | RAA | 6.03 | 4.32 | 5.84 | 5.37 | 4.87 | 2.48 | 4.02 | 2.40 |
|  | RC | 1.36 | 0.97 | 1.322 | 1.21 | 1.10 | 0.56 | 0.91 | 0.54^*^ |
|  | SRC | 86.03 | | | | | | | |
| BB | RAA | 3.1 | 2.69 | 1.84 | 4.38 | 3.03 | 1.8 | 2.79 | 2.9 |
|  | RC | 1.10 | 0.95 | 0.65 | 1.55 | 1.07 | 1.03 | 0.99 | 0.63^*^ |
|  | SRC | 79.50 | | | | | | | |

Note: RAA represent ratio of amino acid, RC represent ratio coefficient of amino acid, SRC represent score of RC. * First limiting amino acid

**Table S3**

The closeness of the three sample proteins relative to the standard protein.

| Amino acid | CC | MM | BB |
| --- | --- | --- | --- |
| Test code | U_1_ | U_2_ | U_3_ |
| Proximity | 0.89 | 0.80 | 0.64 |

**Table S4**

TAV values of amino acids of the three samples.

| Presenting flavors | Amino acid | TAV | | |
| --- | --- | --- | --- | --- |
|  |  | CC | MM | BB |
| Sweetness | Gly | 0.96 | 1.01 | 0.78 |
|  | Ala | 3.17 | 3.3 | 3.24 |
|  | Thr | 1.04 | 1.16 | 0.08 |
|  | Pro | 0.16 | 0.17 | 0.08 |
|  | Cys | 0.13 | 0.15 | 0.40 |
|  | | | | |
| Bitterness | Leu | 1.15 | 1.18 | 1.11 |
|  | Val | 2.11 | 2.62 | 2.24 |
|  | Ile | 0.23 | 0.64 | 0.93 |
|  | Met | 1.08 | 1.21 | 1.11 |
|  | Phe | 0.17 | 0.24 | 0.21 |
|  | Trp | 0.09 | 0.12 | 0.07 |
|  | His | 1.17 | 1.62 | 0.25 |
|  | | | | |
| umami | Glu | 4.01 | 3.24 | 1.72 |
|  | Asp | 4.52 | 4.23 | 2.55 |

**Table S5**

The aroma compounds identified and quantified in three samples.

| Number | Aroma compounds | Formula | Contents of volatile aroma compounds in 3 samples/(μg/g) | | |
| --- | --- | --- | --- | --- | --- |
|  |  |  | BB | MM | CC |
| ***Esters*** | | | | | |
| ES1 | Ethyl palmitate | C_18_H_36_O_2_ | ND | 1.646±0.072 | 1.553±0.063 |
| ES2 | Ethyl caprate | C_12_H_24_O_2_ | ND | 0.629±0.002 | 1.102±0.018 |
| ES3 | Ethyl caprylate | C_10_H_20_O_2_ | ND | ND | 0.531±0.009 |
| ES4 | Ethyl heptanoate | C_9_H_18_O_2_ | 0.366±0.018 | 1.422±0.171 | 1.646±0.001 |
| ES5 | Butyl acetate | C_6_H_12_O_2_ | ND | 0.952±0.012 | 0.920±0.024 |
| ES6 | Ethyl caproate | C_8_H_16_O_2_ | 0.788±0.008 | 1.481±0.029 | 1.422±0.012 |
| ES7 | Ethyl acetate | C_4_H_8_O_2_ | ND | 1.342±0.023 | 1.225±0.041 |
| ES8 | Phenethyl acetate | C10H12O2 | 0.203±0.003 | 0.868±0.013 | 1.680±0.022 |
| ES9 | 2-Ethylhexyl salicylate | C_15_H_22_O_3_ | ND | ND | 0.662±0.035 |
| ES10 | Isoamyl acetate | C_7_H_14_O_2_ | ND | ND | 1.041±0.075 |
| ES11 | Isopentyl isobutyrate | C_9_H_18_O_2_ | ND | 0.541±0.003 | 0.981±0.018 |
|  | ∑ |  | 1.357±0.056 | 8.881±0.352 | 12.249±0.327 |
| ***Alcohols*** | | | | | |
| AL1 | Ethanol | C_2_H_6_O | 1.970±0.131 | 2.816±0.615 | 4.226±0.691 |
| AL2 | 3-Octanol | C_8_H_18_O | 0.611±0.072 | 0.882±0.112 | 0.820±0.011 |
| AL3 | 1-Octen-3-ol | C_8_H_16_O | ND | 6.374±0.802 | 5.031±0.815 |
| AL4 | Phenylethyl alcohol | C_8_H_10_O | 0.793±0.022 | 0.852±0.012 | ND |
| AL5 | 1-Octanol | C_8_H_18_O | ND | 2.215±0.008 | 1.809±0.012 |
| AL6 | n-propanol | C_3_H_8_O | ND | 0.928±0.007 | 0.787±0.007 |

Table S5 Continued

| Number | Aroma compounds | Formula | Contents of volatile aroma compounds in 3 samples/(μg/g) | | |
| --- | --- | --- | --- | --- | --- |
|  |  |  | BB | MM | CC |
| AL7 | 2-butanol | C_4_H_10_O | ND | 0.085±0.015 | 0.065±0.021 |
| AL8 | 2-Nonen-1-ol | C_9_H_18_O | ND | 0.635±0.008 | 0.553±0.013 |
| AL9 | trans-2-Octen-1-ol | C_8_H_16_O | 0.092±0.012 | ND | ND |
| AL10 | n-heptanol | C_7_H_16_O | ND | 0.581±0.012 | 0.558±0.002 |
| AL11 | 3-methylhexan-2-ol | C_7_H_16_O | ND | 0.044±0.051 | 0.068±0.021 |
|  | ∑ |  | 3.466±0.237 | 15.412±1.650 | 13.917±1.477 |
| ***Aldehydes*** | | | | | |
| AD1 | Hexanal | C_6_H_12_O | 0.942±0.002 | 0.973±0.002 | 0.763±0.007 |
| AD2 | Decanal | C_10_H_20_O | 0.164±0.002 | 0.302±0.002 | 0.012±0.002 |
| AD3 | Octanal | C_8_H_16_O | 0.079±0.012 | ND | ND |
| AD4 | Benzaldehyde | C_7_H_6_O | 0.426±0.016 | 1.226±0.013 | 1.421±0.034 |
| AD5 | Furfural | C_5_H_4_O_2_ | 0.008±0.031 | ND | ND |
|  | ∑ |  | 1.691±0.063 | 2.535±0.017 | 2.196±0.043 |
| ***Acids*** | | | | | |
| AC1 | Hexanoic acid | C_6_H_12_O_2_ | 0.265±0.031 | 0.165±0.022 | 0.151±0.003 |
| AC2 | Acetic acid glacial | C_2_H_4_O_2_ | 0.226±0.005 | ND | 0.204±0.001 |
| AC3 | Decanoic acid | C_10_H_20_O_2_ | 0.534±0.022 | 0.334±0.012 | ND |
| AC4 | Butyric acid | C_4_H_8_O_2_ | 0.062±0.005 | 0.726±0.021 | 0.703±0.023 |
|  | ∑ |  | 1.087±0.108 | 1.225±0.055 | 1.058±0.027 |
| ***Alkenes*** | | | | | |
| AK1 | p-cymene | C_10_H_14_ | ND | 0.045 ± 0.005 | 0.019± 0.002 |
| AK2 | 2,6-Dimethylundecane | C_13_H_28_ | ND | 0.006 ± 0.001 | 0.012 ± 0.001 |

Table S5 Continued

| Number | Aroma compounds | Retention time (min) | Contents of volatile aroma compounds in 3 samples/(μg/g) | | |
| --- | --- | --- | --- | --- | --- |
|  |  |  | BB | MM | CC |
|  | ∑ |  | ND | 0.051 ± 0.006 | 0.031 ± 0.003 |
| KE1 | 3-Octanone | C_8_H_16_O | 0.382±0.006 | 0.565±0.003 | 0.528±0.011 |
| KE2 | 2-Nonanone | C_9_H_18_O | ND | 0.170±0.003 | 0.070±0.002 |
| KE3 | 3-Heptanone,6-methyl- | C_8_H_16_O | ND | 0.552±0.004 | ND |
| KE4 | 2-Heptanone | C_7_H_14_O | ND | 0.329±0.007 | 0.354±0.013 |
|  | ∑ |  | 0.382±0.006 | 1.616±0.017 | 0.938±0.026 |
| ***Phenols*** | | | | | |
| PH1 | Phenol | C_6_H_6_O | ND | 3.226±0.015 | 2.265±0.052 |
| PH2 | [Guaiacol](https://www.chemsrc.com/en/cas/90-05-1_1191714.html) | C_7_H_8_O_2_ | 0.085±0.004 | 0.053±0.008 | ND |
| PH3 | 4-Ethylphenol | C_8_H_10_O | ND | 0.830±0.004 | 0.503±0.006 |
|  | ∑ |  | 0.085±0.004 | 4.109±0.027 | 2.768±0.058 |
| ***Sulfur*** | | | | | |
| SU1 | [Dimethyl trisulfide](https://www.chemsrc.com/en/cas/3658-80-8_80208.html) | C_2_H_6_S_3_ | ND | 0.882±0.016 | 0.298±0.002 |
| SU2 | Dimethyl tetrasulfide | C_2_H_6_S_4_ | ND | 1.003±0.004 | 0.562±0.006 |
| SU3 | 2-n-Amylthiophene | C_9_H_14_S | 0.023±0.001 | 0.062±0.003 | 0.084±0.012 |
|  | ∑ |  | 0.023±0.001 | 1.947±0.023 | 0.944±0.020 |
| ***Helerocycle*** | | | | | |
| HE1 | [2-Amylfuran](https://www.chemsrc.com/en/cas/3777-69-3_196939.html) | C_9_H_14_O | ND | 0.672±0.036 | 0.542±0.041 |
| HE2 | Indole | C_8_H_7_N | ND | 3.422±0.071 | 2.824±0.067 |
|  | ∑ |  | ND | 6.094±0.107 | 3.366±0.109 |

Note: “ND” indicated the substance was not detected.

**Fig. S1.** Textural properties of the three samples (*P*<0.05).

**
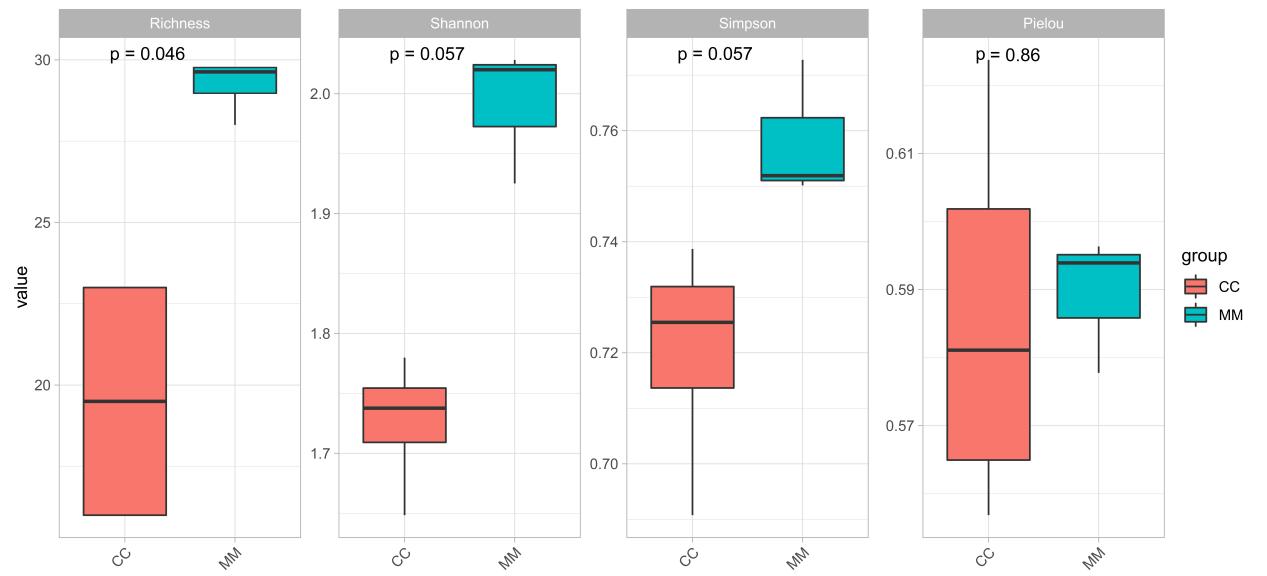
**

**Fig. S2.** The effect of isolation method on alpha. For alpha diversity richness, Shannon,Simpon and Pielou indices are presented.
